# New Test for Sugar in Diabetes

**Published:** 1854-11

**Authors:** John Horsley


*New Test for Sugar in Diabetes.* By JOHN HORSLEY.—If a freely alkaline solution of chromate of potassa be mixed with urine suspected to contain sugar, and boiled, the liquor will assume a deep sap green color, arising from the decompostion of the chromic acid, the reduced oxide of chromium being held in suspension by the potassa.

Such is the sensitiveness of this test that five or six drops only of saccharine urine difused through water is sufficient to show the effect, which is infinitely more striking than even Moore's potassa or Trommer's Test.

I would, therefore, recommend a mixture, in equal parts, of a solution of the neutral chromate of potassa and liquor potassæ, to be kept in the Chemical Cabinet of every chemical practitioner, labelled "Test for Sugar." The following two experiments beautifully illustrate the value of this process in the detection of sugar under any circumstances:

*First Experiment.*—take a small test tube, and having put into it ten or twelve drops of simple syrup (cane sugar) and diluted it with water, add a few drops of the test mentioned, and apply the heat of a spirit lamp. No effect will be produced.

*Second Experiment.*—Take another test tube, and having put the same quantity of simple syrup diluted with water, and two cr three drops of acid, sulph. dil. and boil for a few minutes; this will convert the cane into grape sugar. If we now add a few drops of the test and apply heat, the effect becomes striking, developed in the change of the color of the liquid to an intense green.

When the quantity of sugar is very small, a piece of white paper at the back of the test tube will show the color more distinctly.—*London Chemist.*

